# Prevention of psychosis: moving on from the at-risk mental state to universal primary prevention

**DOI:** 10.1017/S003329172000313X

**Published:** 2021-01

**Authors:** Robin M. Murray, Anthony S. David, Olesya Ajnakina

**Affiliations:** 1 Department of Psychosis Studies, Institute of Psychiatry, Psychology & Neuroscience, King's College London, London, UK; 2 Department of Psychiatry, Experimental Biomedicine and Clinical Neuroscience, University of Palermo, Palermo, Italy; 3 Institute of Mental Health, University College London, London, UK; 4 Department of Biostatistics & Health Informatics, Institute of Psychiatry, Psychology & Neuroscience, King's College London, University of London, London, UK

**Keywords:** At-risk mental state, pathways to care, psychosis, schizophrenia, transition, prevention

## Abstract

The value of services for those with the ‘At Risk Mental State for Psychosis’ (ARMS) continues to be disputed. ARMS services have provided a valuable stimulus to academic research into the transition into psychosis. Furthermore, there is currently a welcome trend to transform such clinics into youth mental health services catering for the broader clientele of young people suffering from anxiety and depression, who already constitute the bulk of those seen at ARMS clinics. However, such services are never likely to make major inroads into preventing psychosis because they only reach a small proportion of those at risk. Evidence from medicine shows that avoiding exposure to factors which increase the risk of disease (e.g. poor nutrition, transmission of infection, tobacco smoking), produces greater public benefit than focussing efforts on individuals with, or about to develop, disease. We consider that the most productive approach for psychosis prevention is avoiding exposure to risk-increasing factors. The best-established risk factors for psychosis are obstetric events, childhood abuse, migration, city living, adverse life events and cannabis use. Some as city living, are likely proxies for an unknown causal factor(s) while preventing others such as childhood abuse is currently beyond our powers. The risk factor for psychosis which is most readily open to this approach is the use of cannabis. Therefore, as an initial step towards a strategy for universal primary prevention, we advocate public health campaigns to educate young people about the harms of regular use of high potency cannabis.

## Introduction

The merits and demerits of services for those with the At-risk Mental State for Psychosis continue to be the subject of heated argument (Malhi, Bell, Hamilton, & Morris, 2020; McGorry & Nelson, 2020; Moritz, Gawęda, Heinz, & Gallinat, 2019; Nasrallah, 2020; Rabello, Poletti, & Preti, 2020; Woods et al., 2020; Yung et al., 2019). Some such as Malhi et al. (2020) suggest that such services in themselves harm. We do not ourselves believe this and consider that the development of such services has undoubtedly provided an academic stimulus as well as the provision of a valuable source of pre-psychotic patients for biomarker research (Ajnakina, David, & Murray, 2018; Green, McGuire, Ashworth, & Valmaggia, 2011; Nasrallah, 2020); the latter has produced a steady stream of interesting findings. For example, brain changes have been documented in ARMS individuals who convert to psychosis compared to those who did not (Cannon et al., 2015; Pantelis et al., 2005; Takahashi et al., 2009; Woods et al., 2020). Also, individuals with ARMS who proceeded to develop clinical psychosis were shown to have an excess capacity to synthesize striatal dopamine, which increased further as they got nearer to clinical psychosis, compared to healthy controls (Howes et al., 2011).

The aim of our previous article (Ajnakina et al., 2018) was simply to point out that despite their best efforts, ARMS services are unlikely to ever reach more than a fraction of those who are truly at risk of going on to develop frank psychosis (Ajnakina et al., 2017; 2018). Thus, most patients referred to the South London ARMS clinic do not in fact have an ARMS, those referred are relatively advantaged, they have better insight into their mental health needs (Lappin et al., 2007), and have more supportive families than patients who develop psychosis without being referred to the ARMS clinic (Ajnakina et al., 2017; 2018). Yung et al. (2019) do not dispute these findings but rather suggest that our experience in South London may be unrepresentative. We accept this possibility and urge other integrated ARMS services serving defined catchment area populations to replicate our study.

Ethnic minorities deserve special mention since they have an especially high risk of developing psychosis (Boydell et al., 2001; Jongsma et al., 2018). We certainly agree with Yung et al. (2019) that providing them with better mental health care may help reduce the risk of subsequent psychotic disorder. The issue is whether ARMS clinics can do this. Again in South London, but independently of us, Byrne et al. (2019) compared the ethnic distribution of patients presenting directly with their first episode of psychosis (FEP) with those who reached ARMS services for psychosis; they concluded that there were fewer people with Black ethnicity and more with White British ethnicity in the Ultra high risk (UHR) group compared with a FEP group. Research from elsewhere in the UK highlights the ARMS services under-detect cases in non-White populations (Morrison et al., 2011) with similar findings from six sites in the USA (Lynch et al., 2016).

Yung et al. (2019) correctly point out that people attending ARMS services can go on to develop a variety of psychosis other than schizophrenia. We agree with this but note that many people who develop their first episode of mania or those who present with a brief psychotic reaction, do so in the context of relatively good previous mental health. Indeed, Shah et al. (2017) who Yung and colleagues quote as validating the clinical high-risk criteria, found that 32% of FEP never experienced any attenuated positive or subthreshold psychotic symptoms prior to developing frank psychosis. Since they do not have an extended prodromal phase, it will never be possible for such patients to be attracted to ARMS clinics before they develop psychosis.

Yung et al. (2019) state: ‘Rather than calling for the dismantling of their service, Ajnakina *et al*. could be advocating for more resources to increase the accessibility and acceptability of their service more widely.’ We did not call for the dismantling of ARMS services. Rather we noted with approval, that McGorry, Yung and their colleagues appear to have accepted the limitations of ARMS clinics, and now adopt a more comprehensive approach by advocating Youth Mental Health Services to care for those with a range of psychiatric disorders (Mei, Killackey, Chanen, & McGorry, 2019). This approach appears to us to be more logical and has many advantages over clinics aiming at the narrower clientele.

Furthermore, we worry about where extra resources for ARMS services would come from. In many of the countries where ARMS services have been established, the funds have come from the overall budget for the care of people with psychosis. This can lead to an impoverishment of the services for people with established psychosis. In some countries such as Australia, the brilliant advocacy of people like Yung and McGorry has persuaded politicians to provide extra resources for ARMS clinics. However, their compatriots, Alison, Bastiampillai, Malhi, and Castle (2019) recently complained that ‘In response to advocacy on early intervention, the Victorian government … spends twice the national per capita average on youth specific mental health services’ while at the same time spending 27% less than the national per capita average on general adult psychiatry. Allison and colleagues ask, ‘whether having world leading youth services, and the nation's lowest investment in general adult services makes sense’.

### A public health approach to prevent psychosis

Yung et al. (2019) are correct to chide us for not considering ARMS services to be part of a public health approach. We accept that ARMS services can be regarded as selective prevention. However, even if ARMS services for psychosis were able to prevent patients from developing a psychotic disorder, the distribution of risk in society would remain largely unaffected by their intervention (McKinlay, 1993). This is because ARMS clinics do not influence the exposures across the population which increase the risk of psychosis. Therefore, we argue that a better approach would be public health interventions with the primary focus on reducing exposure to the risk factors. This approach was famously employed by John Snow in 1854 when he brought a cholera epidemic to an end by taking the handle off the Broad Street water pump. In medicine greater change has often been produced by universal primary prevention which targets the whole population rather than intensive treatment of the individual; for example, minimizing or eradicating infectious diseases by vaccination (e.g. smallpox) or improved hygiene (e.g. COVID19 in the absence of a vaccine) or remedying maternal nutritional deficits, such as folic acid deficiency to avoid neural tube defects. Another approach sets out to achieve lifestyle change. A recent successful example of the latter has been national campaigns against tobacco smoking which have resulted in large reductions in smoking rates and smoking-related diseases.

### Moving on to population-based preventive approaches

A number of authors have raised the possibility of applying the principles of primary prevention to psychiatry in general (Arango et al., 2018) and to depression (Hoare, Callali, & Berk, 2020), but only a few studies have focussed on psychosis. Regarding the latter, both McGrath (2000) and Warner (2001) advocated improving pre-and perinatal care including nutrition, particularly for mothers who had schizophrenia, in the expectation that this would result in fewer babies with early neurodevelopmental damage and consequently predisposed to schizophrenia. In a similar vein, Brown and McGrath (2010) considered the targeting of prenatal infections and nutritional deficiencies. Sommer et al. (2016) took a different tack and pointed to the social and cognitive impairments which typically develop years before the ARMS stage; they recommended that prevention should focus on those children at risk for psychosis by virtue of increased familial/genetic risk or minor psychotic symptoms. They suggested that interventions could aim to improve stress resilience, optimize brain maturation, and prevent or alleviate adverse environmental circumstances. Recently, Anglin, Galea, and Bachman (2020) made a passionate plea for a recalibration of the priorities of US funding institutions towards research into reduced exposure to putative social risk factors for psychosis such as poverty and racism.

We consider that interventions aimed at the whole population would be most fruitful. In medicine, such strategies generally depend on identifying exposures that increase the risk of the disorder in question and then publicly advocating avoidance of that risk factor. What do we know about exposures relevant for psychosis? Studies demonstrating that psychosis varies in incidence might provide some clues as to the exposures of interest. The recent EU-GEI study of 16 sites reported that the incidence of psychosis was more than five times higher in certain of the Northern European cities studied than that in parts of Italy and Spain (Jongsma et al., 2018).

It is implausible to think that the differences in the EU-GEI study are due to genetic differences between Northern and Southern Europeans. Differential exposure to environmental risk factors is much more likely. However, there are a considerable number of candidate factors (Stilo & Murray, 2019). Brown and McGrath (2010) suggested the use of the population attributable fraction (PAF) as a measure which could help to decide which factors were important enough to be selected for preventative effort. The PAF is the estimated proportion of all cases of a disorder which could be prevented if one could completely remove the risk factor under study. In interpreting PAF estimates, it is always worth recalling that because psychosis is multifactorial, adding up PAFs for different component causes can come to over 100%.

Kirkbride et al. (2010) estimated the PAF for urbanicity in England to be 19.3% and that for ethnic minority populations 21.6%; the latter means that if all the factors associated with an increased risk of psychotic illness in ethnic minority populations could be successfully identified and removed from the whole population, up to 21.6% of all cases of could be prevented. Bebbington et al., estimated the PAF for child sexual abuse in England as 14%. Obviously, the PAF will vary geographically depending on the prevalence of the risk factor in that particular area. For example, the PAF for use of high potency cannabis varied in the EUGEI study from less than 2% in Bologna, Italy to 50% in Amsterdam, Holland.

We will now consider the prospects for primary prevention focussing on what we regard as the leading candidates: urbanicity, migration, and cannabis use.

### The example of urbanicity

Although being born or brought up in a city rather than a rural setting is a highly replicated risk factor (Stilo & Murray, 2019), urbanicity must be a proxy for one or more specific component causes. High population density, greater exposure to stress, drug abuse, crime (Newbury et al., 2018) have all been suggested. More recently, novel hypotheses focussing on air pollution (Newbury et al., 2019), lack of green space (Engemann et al., 2018), and lack of social capital (Rotenberg, Anderson, & McKenzie, 2020) have all been investigated.

Is it possible that cities could be designed so that their poor areas are less toxic? Certainly, planning cities to have more green space or lower pollution could benefit not only those at risk of schizophrenia but children from poor families more widely (Centre for urban design & mental health, 2020). However, at present, there is insufficient evidence concerning the exact pathogenic mechanism for us to advocate particular changes in town planning which would diminish the psychotogenicity of cities. Nevertheless, there is an urgent need for careful evaluation of the effects of urban planning on mental health.

### The example of migration/ethnic minority status

One factor which contributed significantly to the difference between the incidence of psychosis in Northern and Southern European sites in the EU-GEI study was the substantial population of people from ethnic minorities in Northern cities, mostly migrants and their children. When migrants/ethnic minorities were removed from the analysis, the incidence of psychosis in South London decreased from 61/100 000 per annum to 45/100 000 per annum (Jongsma et al., 2018). If the increased risk is connected to racism and/or discrimination, which is a plausible hypothesis, then we have yet another reason (if one were needed) to back political change to eradicate them (Morgan & Hutchinson, 2010). Unfortunately, we do not think that as yet we are sure enough of the mechanism underlying the increased risk in migrants and their children (Boydell et al., 2001; Jongsma et al., 2018; Sharpley, Hutchinson, Murray, & McKenzie, 2001) to launch an initiative on this basis.

### The example of cannabis use

Use of cannabis is associated with an increased risk of psychosis. As with the initial reports 60 years ago of a causal association between cigarette smoking and lung cancer, there was initial scepticism about the idea that cannabis use increases the risk of psychosis. Critics suggested that the association is not causal but it is due to concomitant use of other drugs, pre-existing deviant personality, or self-medication for preceding psychiatric or prodromal symptoms. One by one these assertions have been rebutted. The risk increases with the frequency and length of use, and the potency of the cannabis used (Di Forti et al., 2019; Marconi, Di Forti, Lewis, Murray, & Vassos, 2016).

The long-term adverse effects of tobacco and alcohol track the extent of their use. Lung cancer reached epidemic proportions after cigarette smoking spread, and the frequency of alcoholic liver disease waxes and wanes in proportion to changes in population alcohol consumption. Growing evidence suggests that this is also the case for cannabis and psychosis. Boydell et al. (2006) showed that the incidence of schizophrenia doubled in London, UK, between 1965 and 1999, and attributed this in large part to the increased use of cannabis. Hjorthøj, Larsen, Starzer, and Nordentoft (2019) demonstrated that the incidence of cannabis-induced psychosis more than doubled in Denmark between 2006 and 2016. Gonçalves-Pinho, Bragança, and Freitas (2020) who studied hospitalizations in Portugal with a primary diagnosis of psychotic disorder or schizophrenia in the 15 years following the decriminalisation of cannabis in 2001, found that those who received a secondary diagnosis of cannabis use rose from 0.87% in 2000 to 10.60% in 2015. Di Forti et al. (2019) found that the incidence rate of psychosis in 11 areas across five countries in Europe was positively correlated highly (*r* = 0.8) with the prevalence of daily cannabis use in the general population in each site. The population attributable fractions (PAFs) calculated indicated that if high-potency cannabis were no longer available, some 12% of cases of first-episode psychosis could be prevented across the 11 sites, rising to 30% in London and 50% in Amsterdam.

Is the evidence sufficiently persuasive to merit preventative action? Gage, Hickman, and Zammit (2016) who exhaustively scrutinized the epidemiological literature for possible confounding, bias, misclassification, reverse causation and other explanations for the association between cannabis use and later psychosis, concluded that ‘epidemiologic studies provide strong enough evidence to warrant a public health message that cannabis use can increase the risk of psychotic disorders’. We concur. Given the lack of an animal model for psychosis and of the equivalent of painting tobacco tar on mice to demonstrate its carcinogenicity, it is not sensible to wait for absolute proof that cannabis is a component cause of psychosis.

## Conclusion

We conclude that ARMS Clinics are never likely to make major inroads into preventing the majority of people who develop psychosis. An approach which could have a greater effect is to focus on avoiding the risk factors which increase the risk of the illness. Although a number of risk factors have been identified, the one which is most readily open to this preventative approach is the heavy use of high potency cannabis. Therefore, as an initial step towards universal primary prevention, we suggest major campaigns to educate young people about the harms of regular use of high potency cannabis. The current global trend to review the legal status of cannabis raises the possibility that whatever decisions about legalisation are taken, major health education campaigns could be initiated informing the public about the risk of heavy use to mental health (Murray & Hall, 2020).
